# One-Step Ambient-Condition Synthesis of PEG- and PVA-Coated SPIONs: Morphological, Magnetic, and MRI Performance Assessment

**DOI:** 10.3390/nano15241902

**Published:** 2025-12-18

**Authors:** Laura Turilli, Angelo Galante, Franco D’Orazio, Valeria Daniele, Giuliana Taglieri

**Affiliations:** 1 Department of Industrial and Information Engineering and Economics, University of L’Aquila, 67100 L’Aquila, Italy; laura.turilli@graduate.univaq.it (L.T.); valeria.daniele@univaq.it (V.D.); 2 Department of Life, Health and Environmental Sciences, University of L’Aquila, 67100 L’Aquila, Italy; angelo.galante@univaq.it; 3 National Institute for Nuclear Physics (INFN), Gran Sasso National Laboratory (LNGS), 67100 L’Aquila, Italy; 4 CNR-SPIN, c/o Department of Physical and Chemical Sciences, 67100 L’Aquila, Italy; 5 Department of Physical and Chemical Sciences, University of L’Aquila, 67100 L’Aquila, Italy; franco.dorazio@univaq.it

**Keywords:** sustainable and scalable synthesis, iron oxide nanoparticles, SPION, XRD, TEM, AGM, MRI

## Abstract

Superparamagnetic iron oxide nanoparticles (SPIONs) are commonly produced through wet-chemical methods that require high temperature and pressure and involve multiple synthesis steps. Our research group has developed an innovative, sustainable, and patented one-step aqueous synthesis operating at ambient temperature and pressure, enabling the direct production of SPIONs in suspension. In this work, we investigated the extension of this method to obtain polymer-coated SPIONs for biomedical imaging applications. Two water-soluble and biocompatible polymers—poly(ethylene glycol) (PEG) and poly(vinyl alcohol) (PVA)—were selected and prepared into twelve samples varying in polymer concentration and iron precursor molarity. Each formulation was characterized and compared to bare SPIONs synthesized with the same approach using X-ray diffraction (XRD), Fourier transform infrared spectroscopy (FT-IR), transmission electron microscopy (TEM), and alternating gradient magnetometry (AGM). The results confirm that the one-step method yields polymer-coated nanoparticles with a cubic spinel magnetite core. PEG produced spherical, monodisperse particles (10–30 nm) exhibiting superparamagnetic behavior but lower magnetization values (1–5 emu/g). In contrast, PVA-coated nanoparticles showed a morphology dependent on polymer concentration and reagent molarity, while maintaining an average size of ~10 nm and superparamagnetic behavior, with magnetization comparable to bare SPIONs (25–50 emu/g). A preliminary MRI evaluation of a selected PVA-coated sample revealed relaxivity values of r_1_ = 0.12 mM^−1^ s^−1^ and r_2_ = 6.44 mM^−1^ s^−1^, supporting the potential of this synthesis route for imaging-oriented nanomaterials.

## 1. Introduction

Iron oxide nanoparticles have emerged as highly versatile nanomaterials over the past few decades, finding applications in areas ranging from sensors, batteries, and catalysis to environmental remediation and biomedical technologies. This unique combination of magnetic properties, high surface area, safety, and biocompatibility makes them particularly attractive for biomedical applications [1,2,3,4,5]. Among these, Fe_3_O_4_ (magnetite) nanoparticles between 3 and 15 nm in size exhibit superparamagnetism, characterized by single-domain magnetization: they reach saturation under an external magnetic field yet exhibit negligible remanence once the field is removed. This property, together with their biocompatibility, underpins the widespread interest in SPIONs (superparamagnetic iron oxide nanoparticles) for applications, such as MRI contrast enhancement, drug delivery, cell labeling, and magnetic hyperthermia [6,7,8,9,10,11,12,13].

Despite their potential, producing SPIONs suitable for biomedical use remains challenging. Physical synthesis routes—including gas-phase deposition, electron beam lithography, pulsed laser ablation, laser-induced pyrolysis, ball milling, and combustion—often require complex, high-energy equipment and provide limited control over particle size, making them less ideal for clinical applications [14]. Biological approaches, using bacteria, fungi, or plant extracts, are environmentally friendly but often yield particles with poor uniformity and stability [15]. Chemical methods, such as coprecipitation, thermal decomposition, microemulsion, or hydrothermal synthesis, enable better control over particle size and monodispersity—crucial for magnetic and relaxation performance—but typically require high temperatures and multiple purification steps, limiting scalability and increasing costs [14,16,17].

Another critical factor for biomedical use is surface functionalization. Uncoated SPIONs tend to aggregate and can disrupt normal cellular functions at high local concentrations. Coating them with biocompatible polymers mitigates these issues, providing colloidal stability and enhanced biocompatibility. Both synthetic polymers (e.g., PEG, PVA, PVP, PLA) and natural polymers (e.g., dextran, chitosan, polypeptides) have been extensively explored for SPION functionalization [17,18,19,20].

To address these challenges, our group at the University of L’Aquila (Italy) developed a patented, one-step, room-temperature aqueous synthesis of monodisperse Fe_3_O_4_ nanoparticles [21,22,23]. This method operates under ambient conditions, avoids post-synthesis purification, and generates no toxic waste. Building on this platform, we further improved biocompatibility and colloidal stability by coating nanoparticles with hydrophilic polymers. PEG and PVA were selected due to their steric stabilization capabilities and proven efficacy in SPION-based biomedical applications [11,24,25,26].

Beyond improving colloidal stability and biocompatibility, polymer coatings also play a crucial role in determining the magnetic and relaxation properties of SPIONs, which directly affect their performance as MRI contrast agents. SPIONs reduce the longitudinal (T_1_) and transverse (T_2_) relaxation times of nearby water protons, with a stronger effect typically on T_2_, leading to signal attenuation in T_2_-weighted images—a hallmark of superparamagnetic agents [20,27,28]. Relaxivity parameters, r_1_ and r_2_, quantify these effects and provide standard metrics to compare formulations and evaluate the impact of different coatings. Previous studies have shown that both the thickness and chemical nature of polymer coatings can significantly influence SPION relaxivity [29,30,31]. For instance, PEG chain length and thinner PEG coatings substantially increase T_2_ relaxivity, while variations in r_1_ and r_2_ have been demonstrated with different biocompatible polymers such as PEG, PCL, chitosan, and dextran [32,33]. The effect of the polymer layer thickness on relaxivity has been investigated, showing that increasing coating thickness can reduce R_2_ due to the exclusion of water protons from the high-field region near the magnetic core [34].

The scientific literature specifically addressing PVA-coated SPIONs is considerably less extensive than that dedicated to more common coatings, such as PEG or dextran. The existing studies primarily focus on the effects of PVA on colloidal stability and magnetic properties, while quantitative MRI relaxivity data (r_1_ and r_2_) remain limited and often difficult to compare due to differences in measurement conditions [35]. This scarcity of systematic relaxivity reports makes it challenging to assess the actual impact of PVA on MRI performance and highlights the need for controlled studies, such as the one presented here.

Preliminary MRI studies on a representative PVA-coated sample synthesized via our one-step method confirmed the expected predominance of transverse over longitudinal relaxation, consistent with the behavior of superparamagnetic iron oxide nanoparticles reported in the literature. These findings highlight the potential of our sustainable synthesis route to produce polymer-coated SPIONs with relaxation properties compatible with imaging applications and motivate further investigation into the effects of polymer identity and synthesis conditions on MRI performance.

## 2. Materials and Methods

For the synthesis of SPIONs coated with PVA or PEG, the following reagents were considered: iron(III) chloride hexahydrate (FeCl_3_·6H_2_O) and iron(II) chloride tetrahydrate (FeCl_2_·4H_2_O) supplied by Carlo Erba Reagents (Milano, IT); anionic exchange resin, used in OH^-^ form, (R-OH) Euroresin SAG-1 FB, supplied by Sicania Chimica (Catania, IT); polyethylene glycol (PEG) with a length chain of 2000 units (PEG-2000) with a molecular weight of 1900–2200 g/mol, and polyvinyl alcohol (PVA), with a molecular weight of 89,000–98,000 g/mol, both supplied by Sigma Aldrich (St. Louis, MO, USA). The only solvent used was distilled water.

Three weight percentages of polymer in water for each polymer were considered: (a) 25%, 50%, and 75% for PEG, and (b) 0.1%, 0.2%, and 0.3% for PVA. Then, for each kind and amount of polymer, the syntheses were carried out according to two different concentrations of iron (III) chloride in water, 0.1 M and 0.3 M. Therefore, for each polymer, six aqueous suspensions of coated SPIONs were produced, named 1PEGx and 3PEGx (x = 25, 50, 75), or 1PVAy and 3PVAy (y = 0.1, 0.2, 0.3), as summarized in Table 1. In addition, to evaluate the differences arising from the presence of polymers, a synthesis without polymers was performed (sample SPION).

For each synthesis, the polymer was dissolved in water until complete solubilization. Then, the iron chlorides in a molar ratio of 2:1 of Fe (III) and Fe (II) were added. In the obtained solution was then poured an anionic resin in order to produce the Fe_3_O_4_ nanoparticles, according to the following reaction [21]:

(1) 2FeCl_3_ + FeCl_2_ + 8(R-OH) → Fe_3_O_4_ + 4H_2_O + 8(R-Cl)

where R-Cl indicates the resin in Chloride form, occurring after the exchange of OH/Cl.

**Figure 1:**
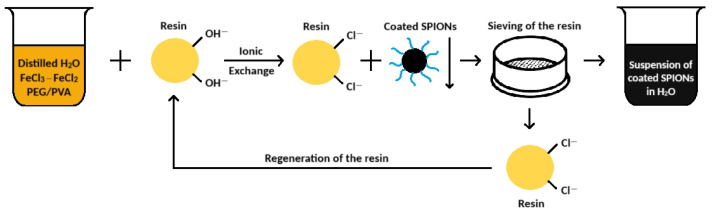
The cyclic process to obtain the coated iron oxide nanoparticles.

The mixing with the anionic resin was carried out under moderate stirring (300 rpm) in air, at room temperature (25 °C) and ambient pressure, for 10 min, according to the procedure previously described [21]. After 10 min, the obtained suspension was separated from the exhaust resin by means of a sieving procedure (mesh < 200 µm). The resin in Cl-form (R-Cl) can be, in turn, regenerated by means of an 8 wt% NaOH aqueous solution, to be reused for another production, according to a cyclic procedure, as shown in Figure 1.

The aqueous suspensions from PEG-SPION and PVA-SPION syntheses were characterized by X-Ray Diffraction (XRD) to analyze crystalline structure and crystallinity. Before analysis, a volume of 0.2 mL was homogenously taken from each suspension and left to dry on a zero-background sample holder. XRD scans were recorded in the range 5–80° 2θ, with a step size of 0.026° 2θ, by a PANalytical X’Pert PRO apparatus (Almelo, The Netherlands), with Cu-Kα radiation, equipped with diffracted-beam optics and a PIXCel 1D detector equipped with a monochromator to reduce noise and fluorescence signals. Each experimental diffraction pattern was elaborated by the High Score Plus softwareversion 4.6a (PANalytical, Almelo, The Netherlands), and crystalline phases were attributed by ICDD and ICSD reference databases. The crystalline domain size was estimated using the Scherrer equation, based on the full width at half maximum (FWHM) of the most intense diffraction peaks.

Infrared spectroscopy FT-IR (Nexus FT-IR, ThermoNicolet, Madison, WI, USA) was performed on the dried suspension samples, both on SPIONs and PEG-SPIONs/PVA-SPIONs, in order to analyze the polymer adsorption on the surface of Fe_3_O_4_ nanoparticles. Pure PEG and PVA FT-IR spectra were acquired as well, in order to check their contribution to the signal.

The iron concentration of the coated samples was determined by inductively coupled plasma–optical emission spectroscopy (ICP-OES 5100, Agilent Technologies, Santa Clara, CA, USA).

The weight of polymer in the obtained samples was measured by thermogravimetric analysis (TGA) with a TG-DTA Linseis L81 (Linseis, Selb, Germany). The samples were heated up to 700 °C at 10 °C/min in a static air atmosphere.

Particle size and morphology were analyzed by transmission electron microscopy (TEM Bright Field, Philips CM100, Philips, Eindhoven, The Netherlands). The samples were prepared by dropping each aqueous suspension on a suitable copper grid, which was then analyzed after the evaporation of water. ImageJ software version 1.54g was used to determine the particle size distribution as well.

Magnetic characterization was carried out by means of PMC Micromag 2900 Alternating Gradient Magnetometer (Lakeshore Cryotronics, Westerville, OH, USA). Measurements of magnetization curves up to 12 kOe (1.2 T) were taken at room temperature.

The evaluation of relaxivity parameters was carried out with a 1.0 T preclinical MRI scanner from Aspect Imaging (Shoham, Israel). For each sample, five vials with distilled water solutions at different concentrations (0.01, 0.02, 0.05, 0.1, 0.2 g/L) were prepared and analyzed along with a reference vial of distilled water. The solenoid radiofrequency coil (6.0 cm inner diameter and 10 cm length) allowed for the insertion of all six vials at the same time. For the evaluation of T_1_, a series of Spin Echo images were acquired, with: Field of View (FOV) of 64 × 48 mm^2^; 1 mm in-plane resolution; 6 mm slice thickness; Echo Time (TE) = 4.1 ms; and 12 different Repetition Time (TR) values (TR = 50, 100, 150, 250, 350, 500, 750, 1000, 1500, 3000, 5000, and 10,000 ms). T_1_ maps were calculated by the exponential signal recovery within each image voxel, and the T_1_ value of each sample was given by the average of the corresponding vial’s voxels. The unavailability of a multi-echo Spin Echo MRI sequence prevented the acquisition of T_2_ relaxation mapping from images. Thus, we used a whole-sample Fast Spin Echo pulse sequence with echo train length equal to 128 and disabled gradients, allowing for CPMG (Car–Purcell–Meiboom–Gill) acquisition of every single vial with TE = 4.4 ms, extracting T2 from the echoes’ amplitude exponential decay. Then, the relaxivity parameters r_1_ and r_2_ were obtained from the linear fittings of 1/T_1_ and 1/T_2_ versus the iron concentration of the vials.

## 3. Results and Discussion

### 3.1. X-Ray Diffraction (XRD)

**Figure 2:**
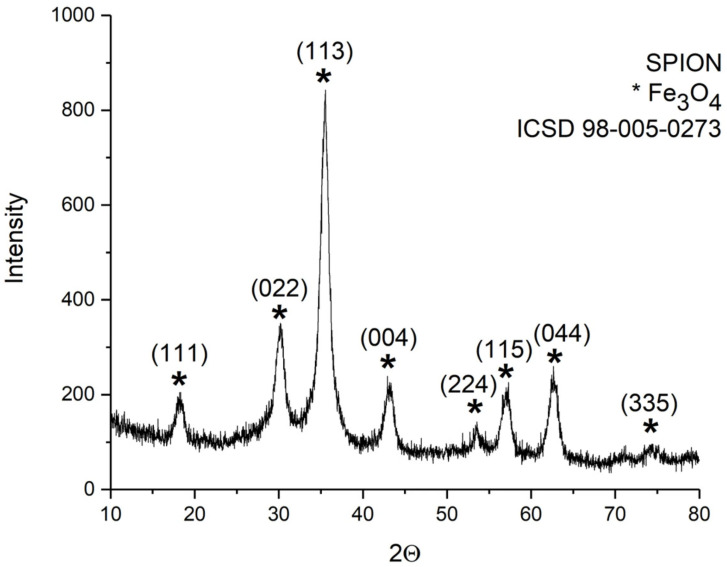
XRD patterns of the dried suspension samples obtained by the synthesis performed without polymers (sample SPION). All the Bragg peaks match the cubic spinel structure of magnetite (ICSD 98-005-0273).

The XRD pattern of the sample SPION is reported in Figure 2. From the analysis, it perfectly matched the cubic spinel structure of magnetite crystalline structure (ICSD standard reference code: 98-005-0273 [36]).

**Figure 3:**
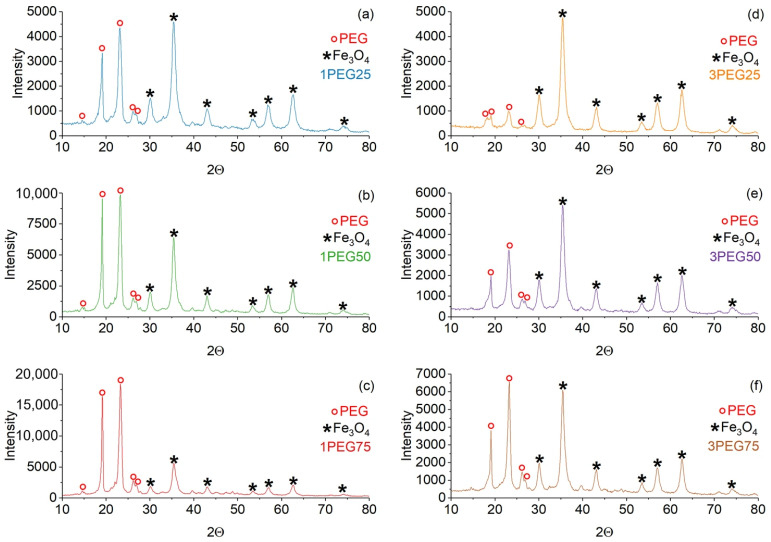
XRD patterns of PEG-SPION dried samples. On the left: (**a**) 1PEG25; (**b**) 1PEG50; (**c**) 1PEG75. On the right: (**d**) 3PEG25; (**e**) 3PEG50; (**f**) 3PEG75. The red circle marker represents the PEG peaks, while the black star marker represents the Fe_3_O_4_ peaks.

**Figure 4:**
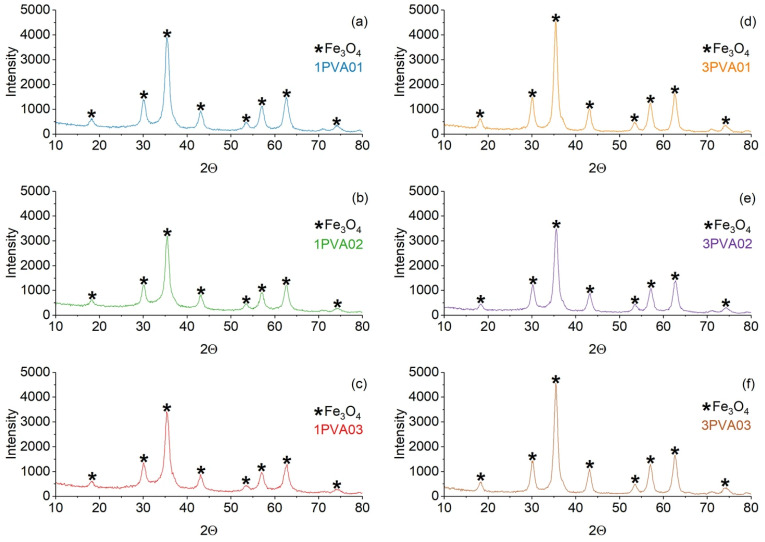
Comparison of the XRD patterns of the PVA-SPION samples. On the left: (**a**) 1PVA01; (**b**) 1PVA02; (**c**) 1PVA03. On the right: (**d**) 3PVA01; (**e**) 3PVA02; (**f**) 3PVA03. The black star marker represents the Fe_3_O_4_ peaks.

XRD patterns of PEG-SPIONx and PVA-SPIONy samples are reported in Figure 3 and Figure 4, respectively. In both samples, the magnetite Bragg peaks are quite recognizable and match the ICSD standard pattern. Since high-chain-length PEG is semicrystalline, the peaks that match the standard pattern of PEG (ICDD 00-049-2095) are also present in the PEG-SPIONx samples [37].

Average crystallite sizes of the magnetite phase, calculated by the Scherrer formula, of each sample are reported in Table 2. From the results, we can observe that the PVA-SPIONy samples show the lowest values of average crystal size of the magnetite core (4.8–6.6 nm) with respect to PEG-SPIONx samples (8.3–10.4 nm). Remarkably, the PVA-SPIONy samples show a similar range to that of the bare SPIONs [21,22].

### 3.2. FT-IR Analyses

FT-IR analysis of SPION samples revealed the characteristic absorbance spectrum of the Fe-O bond in nano-magnetite, at 405 and 418 cm^−1^ (split of ν_1_ band) and 545 cm^−1^ (ν_2_ band) [38,39]. The bare PEG and PVA samples show their typical infrared absorbance peaks. Specifically, the bare PEG sample demonstrated the following: the CH out-of-plane bending vibrations at 947 cm^−1^ and 2871 cm^−1^; the CH_2_ vibrations at 1271 cm^−1^ and 1456 cm^−1^; and the C-O-C ether stretch and vibrations at 883 cm^−1^, 1138 cm^−1^, and 1351 cm^−1^ [38,39,40]. In contrast, the bare PVA sample exhibited the following: the CH deformation and stretching vibrations at 1317 cm^−1^ and 2904 cm^−1^, and the C-O stretching vibrations at 1090 cm^−1^ [41,42]. In addition, in all these samples, a broad band of absorbance around 3200–3500 cm^−1^ reveals the presence of hydroxyl groups attached to the surface [39].

**Figure 5:**
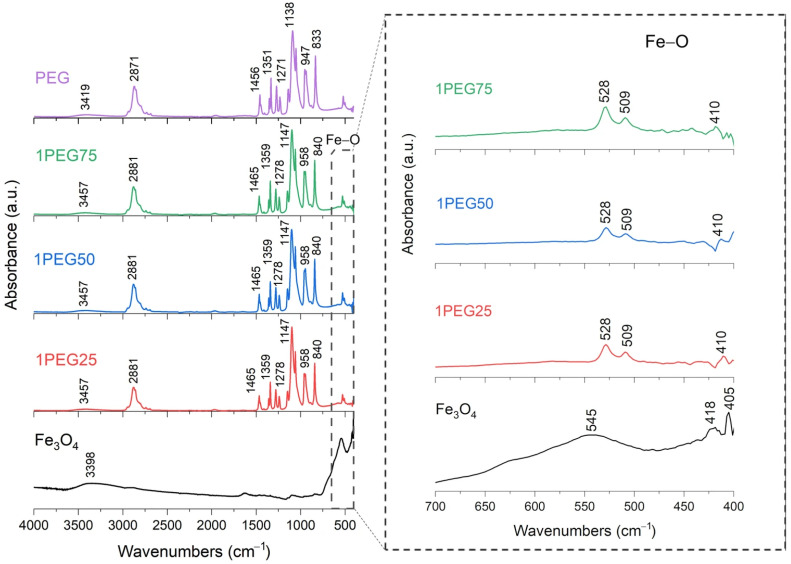
FT-IR analysis of 1PEG25, 1PEG50, 1PEG75 samples. In the inset: the absorption bands of the Fe-O bond at 545 cm^−1^, to underline the shift towards lower wavenumbers and the splitting characteristics of PEG-SPION samples.

**Figure 6:**
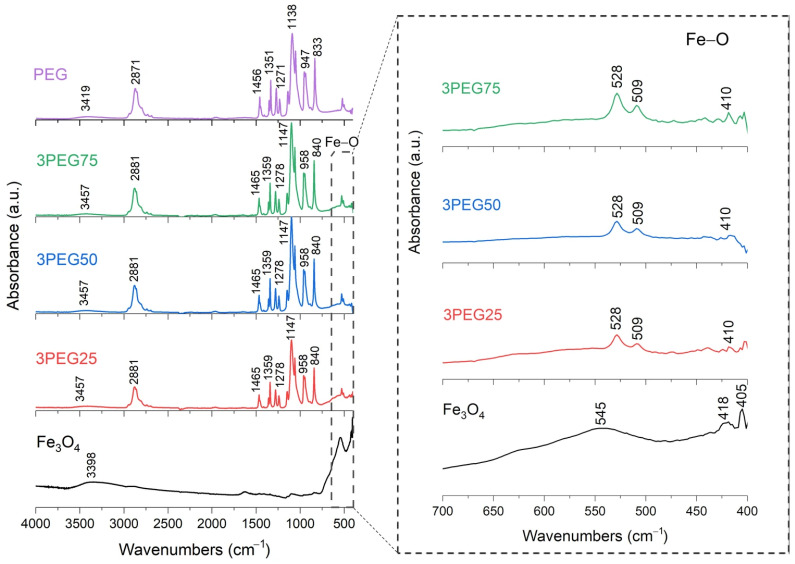
FT-IR analysis of 3PEG25, 3PEG50, 3PEG75 samples. In the inset: the absorption bands of the Fe-O bond at 545 cm^−1^, to underline the shift towards lower wavenumbers and the splitting characteristics of PEG-SPION samples.

The series of PEG-SPIONx samples is reported in Figure 5 and Figure 6. From these results, the Fe-O broad peak at 545 cm^−1^, originally observed in the SPION sample, is split into two sharper peaks, at 508 cm^−1^ and 528 cm^−1^. This change could indicate a change in the environment and the formation of bonds between the Fe_3_O_4_ and the PEG [38,39]. For the same reason, we find that the C bonds of PEG and the Fe-O bond of magnetite are present in all the PEG-SPION samples, but with a little shift.

**Figure 7:**
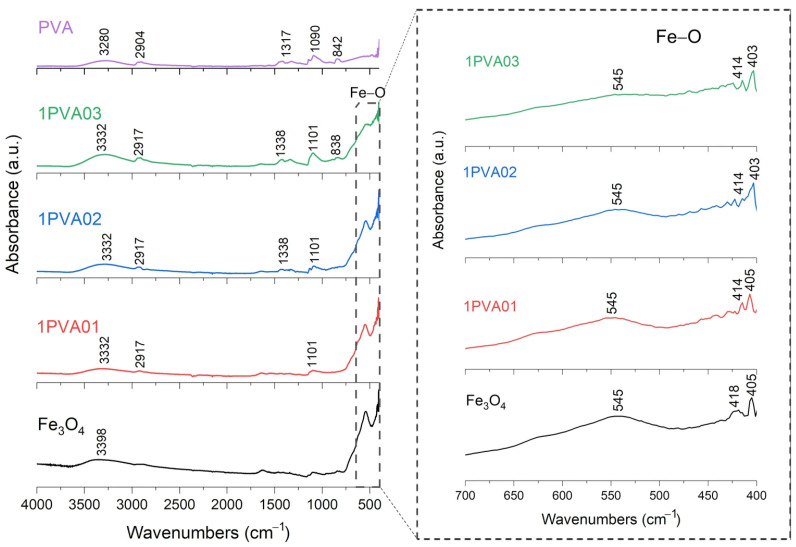
FT-IR analysis of 1PVA01, 1PVA02, 1PVA03 samples. In the inset: the absorption bands of the Fe-O bond at 545 cm^−1^, to underline the shift towards lower wavenumbers and decreasing signal characteristics of PVA-SPION samples.

**Figure 8:**
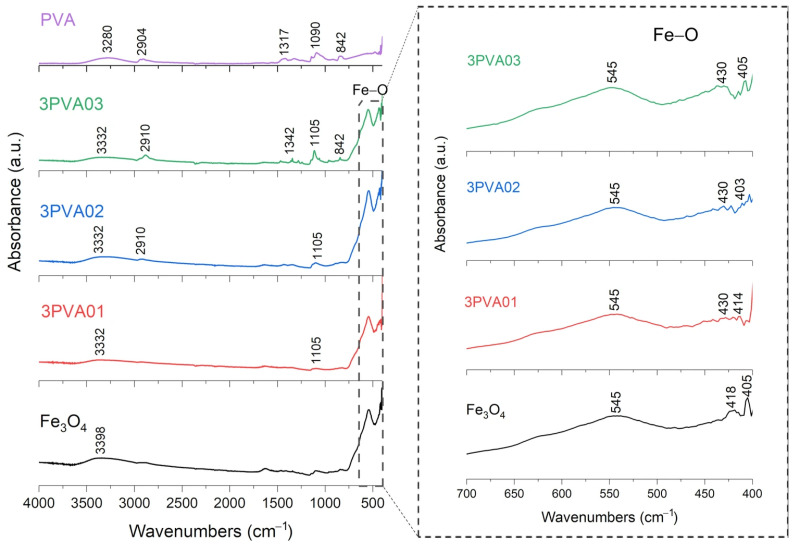
FT-IR analysis of 3PVA01, 3PVA02, 3PVA03 samples. In the inset: the absorption bands of the Fe-O bond at 545 cm^−1^, to underline the shift towards lower wavenumbers and decreasing signal characteristics of PVA-SPION samples.

FT-IR results for PVA-SPIONy samples are shown in Figure 7 and Figure 8. In this case, a decrease in the absorbance band related to the Fe–O bond is observed at increasing PVA concentrations due to the formation of bonds between Fe_3_O_4_ and PVA [42].

To summarize, these FT-IR results reveal distinct interactions between Fe_3_O_4_ and each polymer. For the PEG-SPIONx sample, the splitting of the Fe-O band suggests a modification of the local environment around the Fe–O bonds, and PEG-specific C bands remain present but exhibit slight shifts; in contrast, PVA-SPIONy shows a decrease in the Fe-O band absorbance with increasing PVA. Overall, these observations imply that PEG coating modifies the Fe–O environment while maintaining its spectral visibility, whereas PVA coating results in the partial suppression of Fe–O vibrations.

### 3.3. ICP-OES Analyses

The iron concentration of the polymer-coated samples was determined by ICP-OES. The results showed that the mass percentages of Fe to the total weight of the coated samples were under 50% for the PEG-SPIONx series and above 55% for the PVA-SPIONy series. These results indicate that for the PEG-SPIONx samples, the quantity of magnetite, in weight, is lower than that of the PVA-SPIONy samples, probably due to the higher concentrations of PEG used.

The exact percentages for all samples are reported in Table 3.

### 3.4. TGA

The amount of polymer in each sample was determined by TGA, measured as the loss of weight of every sample at the end of the analysis, which was conducted up to 700 °C to ensure the total combustion of the polymer [38,41]. The polymer amount for the PEG-SPIONx series was more than 50% for all samples, while for the PVA-SPIONy series, the polymer amount was under 10%. These results confirm that PEG-SPIONx samples contain, in weight, lower amounts of magnetite than PVA-SPIONy, as seen in the ICP-OES analyses.

The exact percentages for all samples are reported in Table 4.

### 3.5. TEM Analyses

**Figure 9:**
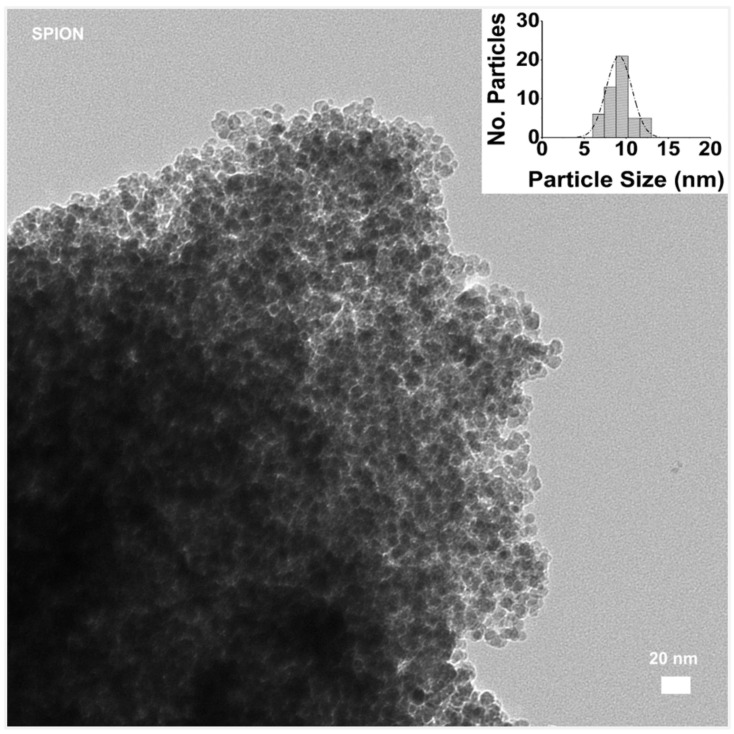
TEM image of SPION sample without polymer addition, bar size 20 nm. (In the inset, the distribution of particle size evaluated with ImageJ software).

The morphologies and sizes of the prepared polymer-modified magnetite were analyzed by TEM. For this aim, at first, we observed the prepared magnetite (Fe_3_O_4_) NPs alone, as shown in Figure 9. Both the morphology and monodispersed distribution of the magnetite particles offer an important starting point, allowing for a uniform surface area for coating and conjugation of targeting ligands or therapeutic agents.

**Figure 10:**
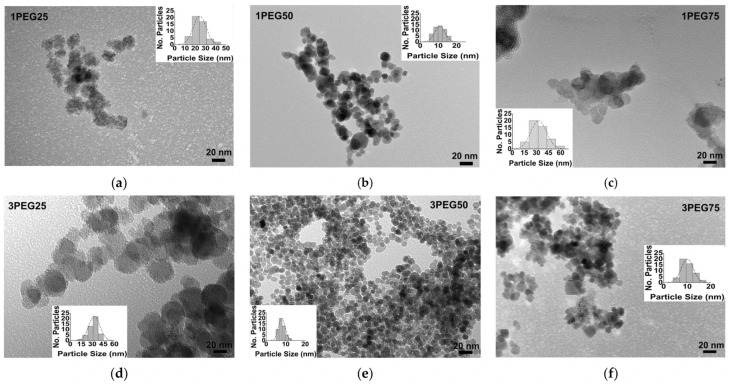
TEM images of PEG-SPIONx samples: (**a**) sample 1PEG25, bar size 20 nm; (**b**) sample 1PEG50, bar size 20 nm; (**c**) sample 1PEG75, bar size 20 nm; (**d**) sample 3PEG25, bar size 20 nm; (**e**) sample 3PEG50, bar size 20 nm; (**f**) sample 3PEG75, bar size 20 nm. The evaluation of the size distribution of the particles with ImageJ software is reported in the inset of every figure.

**Figure 11:**
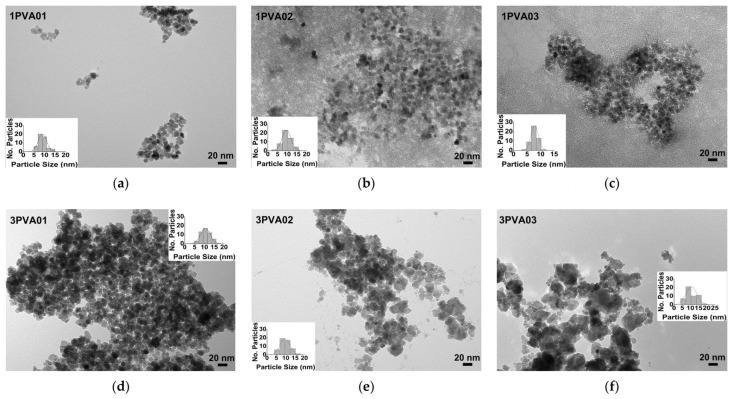
TEM images of PVA-SPIONy samples: (**a**) sample 1PVA01, bar size 20 nm; (**b**) sample 1PVA02, bar size 20 nm; (**c**) sample 1PVA03, bar size 20 nm; (**d**) sample 3PVA01, bar size 20 nm; (**e**) sample 3PVA02, bar size 20 nm; (**f**) sample 3PVA03, bar size 20 nm. The evaluation of the size distribution of the particles with ImageJ software is reported in the inset of every figure.

TEM images of the PEG-SPIONx and PVA-SPIONy samples allowed for evaluating how morphologies and particle sizes changed with the addition of polymers, as shown in Figure 10 and Figure 11, respectively.

The TEM images revealed a spherical morphology of all PEG-SPION particles, independent of the different synthesis conditions, revealing an equiaxial homogeneous coverage of each particle. However, the particles appeared joined to one another, except for the 3PEG50 sample (Figure 10e). In addition, these observations showed a strong influence of PEG amounts and molar concentration of the iron aqueous solutions (0.1 M or 0.3 M) on the particle’s dimensions. Specifically, at a low polymer amount and at the highest iron molar concentration, an increase in particle size was observed; as the polymer amount increased, a slight decrease in dimension was visible at the highest iron molar concentration. The combination of 50% PEG and the highest iron molar concentration (0.3 M) achieved a monodispersed distribution of PEG-SPIONs, less than 10 nm in size.

The observation of PVA-SPIONs samples (Figure 11) showed similar particle sizes when the lowest iron molar concentration and the lowest PVA concentration were considered, with spherical particles having a monodispersed distribution. On the contrary, in the 1PVA02 sample and 1PVA03 sample (Figure 11b,c), the particles appeared surrounded by uniform polymer distribution, as also observed in previous studies, indicating that, at this molar concentration of the reagents, the presence of PVA may promote the growth of colloidal nanocrystal clusters (CNCs) [43]. In the 3PVA02 and 3PVA03 samples (Figure 11e,f), similar agglomerates of overlapping pseudohexagonal particles were observed, underlining a well-defined influence of the polymer concentration on particle morphology.

**Figure 12:**
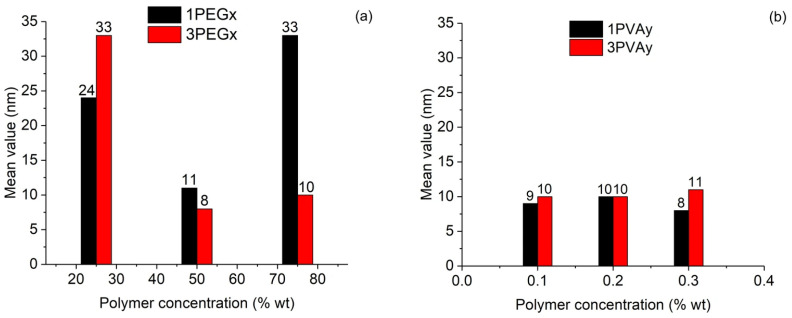
Influence of polymer concentration on size dimension of particles: (**a**) PEG-SPIONs samples; (**b**) PVA-SPIONs samples.

The average values of particle size, with the respective standard deviations, are reported in Table 5. A graphic visualization of the change in particle size due to the variation in polymer concentration is shown in Figure 12.

From these results, we can observe that, when PEG was considered, strong deviations from the 50 wt% addition increased the particle dimensions obtained at low iron concentration. On the contrary, high iron concentration allowed for the particle size to remain around 10 nm only if PEG addition was equal to or higher than 50 wt%. In the case of a PVA addition, a more stable particle size around 10 nm was observed, with a slight average decrease when a low iron concentration was considered.

### 3.6. AGM

**Figure 13:**
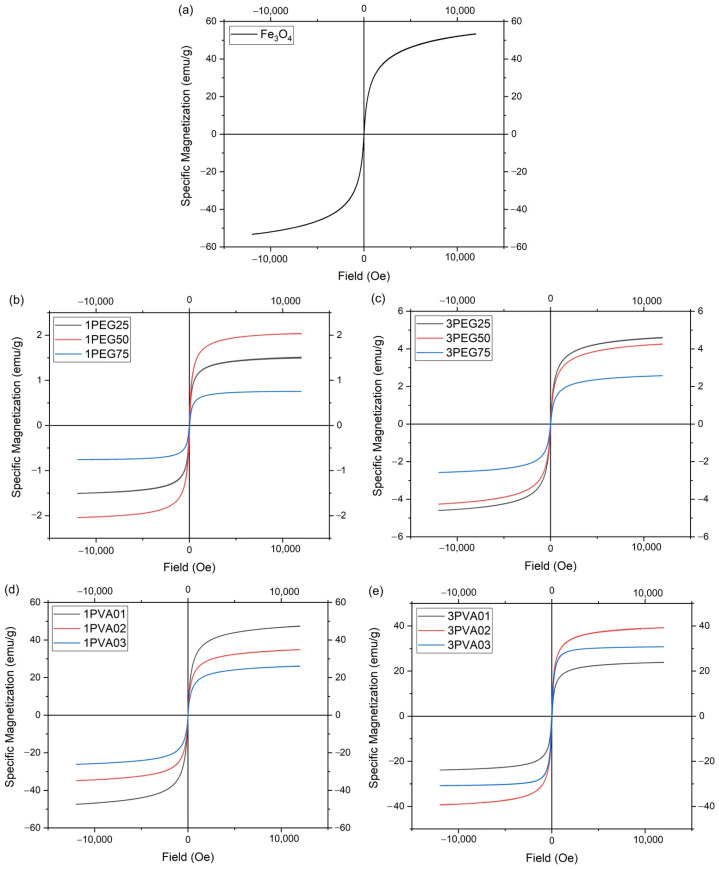
AGM measurements: (**a**) bare SPIONs; (**b**) 1PEGx; (**c**) 3PEGx; (**d**) 1PVAy; (**e**) 3PVAy.

The AGM measurements are reported below (Figure 13), with specific magnetizations of the samples normalized using the total mass of the samples.

The 1PEG and 3PEG samples (Figure 13b,c) showed no hysteresis loop at each concentration, which meant that the particles exhibit superparamagnetic behavior. The 1PEG50 sample exhibited the highest magnetization value of the 1PEG series, while the 3PEG series had a decrease in the magnetization value across increasing polymer concentrations. The 3PEG series had higher magnetization values than the 1PEG series, but all the samples had a much lower magnetization value than the bare magnetite nanoparticles produced with the patented method [21,22]. This can be explained in part by the low ratio of Fe, as seen by ICP-OES analysis, and the great fraction of PEG present in the samples, as seen by the TGA, as the magnetic saturation of magnetite nanoparticles decreased significantly with the increasing coating thickness of the non-magnetic layer [38], in part due to other causes. For example, the contamination and deterioration of surface spins of the magnetite core caused by the polymer coating, as seen in similar cases [37].

The 1PVA and 3PVA series of samples (Figure 13d,e), like all the other samples, showed no hysteresis loop at each concentration, confirming the presence of superparamagnetic behavior. The sample 1PVA01 was the one with the maximum magnetization value of the 1PVA series, very close to the value of the bare nanoparticles. In the 3PVA series, the curve for the 3PVA03 sample had a large initial slope (the so-called initial susceptibility), which indicates a larger average particle size with respect to the other 3PVA samples.

Table 6 shows the value of magnetic saturation (Ms), magnetic remanence (Mr), the ratio Mr/Ms, and coercivity for all samples.

### 3.7. MRI Acquisitions

**Figure 14:**
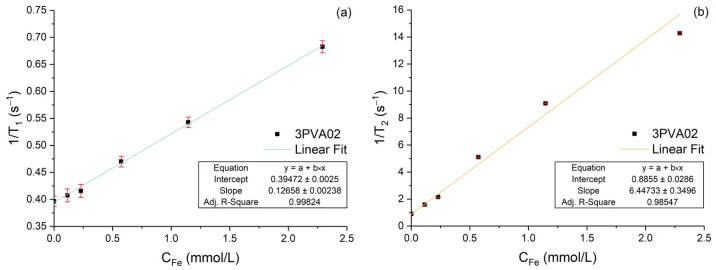
Relaxation rates versus iron molar concentration of the sample 3PVA02: (**a**) longitudinal (1/T_1_), (**b**) transverse (1/T_2_). The lines are the data linear fits, and the insets report the relaxivity values as well as the regression coefficients.

This section reports on the evaluation of relaxivity parameters for the 3PVA02 sample. As shown in Figure 14a, r_1_ is equal to 0.126 ± 0.002 mM^−1^s^−1^ with the regression coefficient of the linear fit R^2^ = 0.998. As shown in Figure 14b, r_2_ is equal to 6.45 ± 0.35 mM^−1^s^−1^ with the regression coefficient of the linear fit R^2^ = 0.985. As expected from the literature [27,29], the particles are more effective in reducing the T_2_ than the T_1_.

These results, combined with the small dimensions of the particles (around 10 nm, as seen from Table 5), and the excellent biocompatibility and biodegradability of polyvinyl alcohol (PVA) in human tissues, demonstrate the potential of this innovative synthesis method to generate coated SPIONs suitable for MRI applications, especially in in vivo imaging [20].

## 4. Conclusions

Superparamagnetic iron oxides remain an important subject of research due to their unique magnetic properties, particularly for biomedical applications. The physical, chemical, and biological routes commonly used to obtain these nanoparticles offer various advantages and disadvantages, often making large-scale production challenging.

Our research group at the University of L’Aquila developed a patented, sustainable, one-step, and scalable synthesis method for producing monodisperse SPIONs, offering a promising solution to these limitations. In this work, we investigated whether polymer-coated SPIONs could be produced directly during synthesis while preserving the one-step nature of the process. Water-soluble polymers, poly(ethylene glycol) (PEG) and poly(vinyl alcohol) (PVA), were selected for their compatibility with the patented method and their well-established use in pharmaceutical and biomedical applications.

Twelve reactions were carried out—six for each polymer—by varying the polymer concentration and the concentration of iron chloride reagents. The resulting twelve samples were analyzed and compared with bare SPIONs using X-ray diffraction (XRD), infrared spectroscopy (FT-IR), inductively coupled plasma–optical emission spectroscopy (ICP-OES), transmission electron microscopy (TEM), and magnetometry (AGM). The XRD results showed that the addition of polymer did not alter the cubic spinel crystal structure of the magnetite core. The FT-IR spectra confirmed successful bonding of PEG and PVA to the surface of the magnetite nanoparticles. The TEM images of PEG-SPIONx samples revealed spherical particles with homogeneous equiaxial coatings, whereas PVA-SPIONy samples exhibited morphologies that varied with iron chloride molarity and PVA concentration, ranging from monodispersed particles to colloidal nanocrystal clusters. The AGM measurements confirmed the superparamagnetic behavior of all samples, with the PVA-SPIONy series showing higher magnetization values. The MRI relaxivity measurements demonstrated a clear enhancement in T_2_ relaxation.

Importantly, while PEG is widely reported in the literature as a coating material for SPIONs, studies employing PVA as a surface modifier remain comparatively limited. Our findings, therefore, contribute valuable insight into the structural, magnetic, and MRI-relevant properties of PVA-coated SPIONs, helping address a gap in the current knowledge regarding this less-explored polymer.

In conclusion, polymer-coated SPIONs can be successfully synthesized using a sustainable, one-step, and scalable method. These nanoparticles show promising potential for biomedical applications, particularly as T_2_ contrast agents for MRI, and offer new perspectives for the use of PVA in SPION surface engineering.

## Figures and Tables

**Table 1:** Summary of the syntheses, in relation to PEG and PVA polymers and iron concentrations.

Sample Series Name	Type of Polymer	Polymer Concentration (wt%)	Iron (III) Chloride Concentration	Sample Name
PEG-SPIONx (x = 25, 50, 75)	PEG-2000	25%	0.1 M	1PEG25
			0.3 M	3PEG25
		50%	0.1 M	1PEG50
			0.3 M	3PEG50
		75%	0.1 M	1PEG75
			0.3 M	3PEG75
PVA-SPIONy (y = 0.1, 0.2, 0.3)	PVA	0.1%	0.1 M	1PVA01
			0.3 M	3PVA01
		0.2%	0.1 M	1PVA02
			0.3 M	3PVA02
		0.3%	0.1 M	1PVA03
			0.3 M	3PVA03

**Table 2:** Average crystallite size of magnetite core calculated by Scherrer equation.

Sample	Average Crystallite Size (nm)
1PEG25	8.3
1PEG50	10.4
1PEG75	8.9
3PEG25	8.5
3PEG50	9.2
3PEG75	9.8
1PVA01	5.6
1PVA02	5.5
1PVA03	4.8
3PVA01	6.6
3PVA02	6.2
3PVA03	6.5

**Table 3:** Mass percentages of Fe in the PEG-SPIONx and PVA-SPIONy samples.

Sample	Mass Percentages of Fe (*w*/*w*%)
1PEG25	46
1PEG50	7
1PEG75	5
3PEG25	26
3PEG50	32
3PEG75	43
1PVA01	81
1PVA02	80
1PVA03	77
3PVA01	63
3PVA02	64
3PVA03	55

**Table 4:** Polymer amount percentages of PEG-SPIONx and PVA-SPIONy samples.

Sample	Polymer Amount (%)
1PEG25	52
1PEG50	71
1PEG75	74
3PEG25	45
3PEG50	50
3PEG75	55
1PVA01	3
1PVA02	6
1PVA03	8
3PVA01	4
3PVA02	9
3PVA03	3

**Table 5:** Mean values of particles’ dimensions evaluated from TEM images at low magnification.

Sample	Mean Value of the Dimension of the Particles (nm)	Standard Deviation (nm)
1PEG25	24	±6
1PEG50	11	±3
1PEG75	33	±10
3PEG25	33	±8
3PEG50	8	±2
3PEG75	10	±3
1PVA01	9	±2
1PVA02	10	±2
1PVA03	8	±1
3PVA01	10	±2
3PVA02	10	±2
3PVA03	11	±3

**Table 6:** Value of magnetic saturation, magnetic remanence, their ratio, and coercivity of all samples.

Sample	M_s_ (emu/g)	M_r_ (emu/g)	Mr/Ms	Coercivity (Oe)
1PEG25	1.52	0.13	0.088	22.40
1PEG50	2.04	0.17	0.085	26.60
1PEG75	0.76	0.05	0.069	20.38
3PEG25	4.61	0.54	0.011	3.77
3PEG50	4.26	0.36	0.010	2.98
3PEG75	2.58	0.11	0.004	1.43
1PVA01	47.43	0.54	0.011	3.77
1PVA02	34.89	0.36	0.010	2.98
1PVA03	26.14	0.11	0.004	1.43
3PVA01	23.88	0.14	0.030	12.30
3PVA02	39.28	0.09	0.021	8.91
3PVA03	30.79	0.08	0.030	13.38

## Data Availability

The original contributions presented in this study are included in the article. Further inquiries can be directed to the corresponding author.
